# Comparison of Piezoresistive Monofilament Polymer Sensors

**DOI:** 10.3390/s140101278

**Published:** 2014-01-13

**Authors:** Mark Melnykowycz, Birgit Koll, Dagobert Scharf, Frank Clemens

**Affiliations:** EMPA Dübendorf, Überlandstrasse 129, Dübendorf 8600, Switzerland; E-Mails: Koll.Birgit@gmail.com (B.K.); Dagobert.Scharf@empa.ch (D.S.)

**Keywords:** sensor, polymer, piezoresistive, high strain, wearable computing, monofilament

## Abstract

The development of flexible polymer monofilament fiber strain sensors have many applications in both wearable computing (clothing, gloves, *etc.*) and robotics design (large deformation control). For example, a high-stretch monofilament sensor could be integrated into robotic arm design, easily stretching over joints or along curved surfaces. As a monofilament, the sensor can be woven into or integrated with textiles for position or physiological monitoring, computer interface control, *etc*. Commercially available conductive polymer monofilament sensors were tested alongside monofilaments produced from carbon black (CB) mixed with a thermo-plastic elastomer (TPE) and extruded in different diameters. It was found that signal strength, drift, and precision characteristics were better with a 0.3 mm diameter CB/TPE monofilament than thick (∼2 mm diameter) based on the same material or commercial monofilaments based on natural rubber or silicone elastomer (SE) matrices.

## 1. Introduction

The evolution of mobile technologies and connected devices are opening new opportunities for the realization of wearable computing applications. However, many wearable devices still rely on traditional sensor types including MEMS-based accelerometers, pressure sensors, gyroscopes, *etc*. These sensors are largely developed as self-contained integrated circuits. To expand the possibilities of wearable computing products, it would be valuable to additionally have stretchable sensors that can conform to the form of the human body [1], and unobtrusively measure force or strains related to human movement. Basing wearable sensor designs on polymers is very interesting because they can be produced in different physical forms, from flexible coatings [2] and sheets [3] to yarns [4], which can be woven into or integrated with textiles for position or physiological monitoring, computer interface control, *etc*. This allows tailored sensor design for different positions on the human body for specific applications.

Previous research into conductive polymers and piezoresistive monofilament fibers has evolved from metallic thread woven with traditional textile materials [5] to the integration of carbon or graphene nano-particles [6] or carbon nanotubes [7,8] into polymer matrices [9], which can then be extruded into monofilament or pressed in sheet forms to create sensor coatings for substrate materials [9]. Silver-plated nylon 66 yarn [10] has also been used to design a wearable sensor for knee angle measurements.

In the current work a piezoresistive polymer monofilament sensor based on carbon black (CB) combined with a thermoplastic elastomer (TPE) [11] was compared with commercially available monofilament fibers produced by Merlin Systems Corp. Ltd. (Plymouth, UK,) and Images SI Inc. (Staten Island, NY, USA).

### 1.1. Sensor Characteristics

Wearable computing presents many challenges for sensor design and packaging. To be effective, sensors need good repeatability and signal stability with low drift. Often these characteristics are tied to environmental factors such as temperature and humidity. In wearable computing applications sensors would be integrated into clothing or other garments, which may be subject to abrasion and high mechanical deformations. Packaging and protection of the sensors is therefore difficult, as compared with micro-electrical mechanical systems (MEMS) applications, where sensor elements can often be packaged and protected.

As a strain sensor for wearable computing, the need is to have a flexible sensor, which changes shape with the body it is placed upon (torso, arm, leg, *etc*.), and produces a reproducible change in resistance in response to mechanical strain, ideally in a linear way for easy integration into a system design. Furthermore, the signal must be reliable over the lifecycle of the product it is used in with regards to signal stability, aging, drift, *etc.* after subjected repeated mechanical cyclic loads. Conventional tensile strain sensors have an operating range of a few percent, with large strain variations attaining 10% strain as with the HBM LD20 high strain gauge [12]. In the conventional metal strain gauge design, a change in electrical conductivity in a thin wire or foil in response to mechanical elongation is measured to determine the strain on the structure being investigated [13]. The performance of strain sensors is generally reported by referencing the gauge factor (*k*) according to Equation (1):

(1) $$\frac{\Delta R}{R}=k\frac{\Delta l}{l}$$

where *R* is the resistance at a strain of zero, *l* is the length at a strain of zero, while DR and Dl are the changes in resistance and length due to an applied mechanical strain. The gauge factor (*k*) for conventional strain gauges is generally close to 2 [12]. Beyond the mechanical strain limit of the sensor the resistance signal becomes unstable due to excessive strain of the sensing element and eventual mechanical failure of the structure occurs. In the current application, a strain of 20%–100% is required, and for this reason traditional strain sensors could be not be employed. Polymer-based materials would be able to fulfill the mechanical deformation requirements. Monofilament fiber strain sensors are an ideal form for wearable computing applications since they can be integrated into clothing in an unobtrusive way. Piezoresistive materials are often used in sensor designs where relationships between deformation and electrical resistance can be characterized and used in circuit design. However, research into piezoresistive sensors has mainly focused on the doping of silicon structures, and not on larger scale flexible sensors. Flexible elastomeric sensors can be achieved by using dielectrics and electro-active polymers (EAPs), elastomers with carbon-based electrodes have been investigated, but generally with the desire to develop flexible force actuators. Alternatively, a conductive polymer composite (CPC) can be created by combining a conductive filler (e.g., silver, carbon nanotubes, carbon black) and a polymer matrix (elastomer, thermoplastic, *etc.*).

### 1.2. Elastomer Capacitor Sensor

EAPs based on elastomers coated with conductive layers have been investigated for flexible force actuator applications. In a sensor configuration, highly flexible capacitive sensors can be created [14]. A problem though is that capacitive sensing is based on the assumption that the gap between electrodes will change, while the electrical boundary conditions of the electrodes will remain constant [15]. However, shape changes in terms of tensile strain will lead to a change in conductivity of the electrodes in addition to the change in electrode separation, making the material combination complicated to characterize as a simple strain sensor. For example, if a parallel capacitive sensor is designed using a flexible electrode, the electrode area increases as the sensor is compressed. Then the sensor signal changes due to the decrease in gap distance between the electrodes, as well as due to the change in resistance of the electrode geometry changing [14,16]. With dielectric elastomers one primary research goal is the development of compliant electrodes, where a change in mechanical dimension does not lead to a large change in resistivity of the material [17]. This will be very useful for flexible circuit applications, but is not useful at this time for tailoring a high-stretch strain sensor, where a change in resistivity of the material with respect to applied mechanical deformation is required [9,18–20].

### 1.3. Piezoresistive Polymer Sensors

Different types of piezoresistive sensors have been developed over the past decade with a focus on strain or force-sensing applications. Historically flexible fiber sensors have followed different directions including the development of yarns with conductive fibers [5,21] or nanotube yarns [22], electroactive polymers with carbon fillers [9,19], carbon or silver-coated polymer fibers [10] and the integration of carbon nanotubes into a polymer matrix [23]. The yarn design includes a sensor fiber element woven or twisted together with structural but electrically insulating fibers. The conductive fiber could be metallic or carbon-coated [5,24]. This combination allows the integration of sensing properties into a structural package, where the sensor material can support mechanical loads and provide a signal (while traditional strain gauges only provide passive sensing of substrate deformation). The basic design of a piezoresistive sensor monofilament entails combining conductive particles together with a flexible polymer matrix, creating a CPC material. The material can then be formed into various forms from monofilaments to plates for specific applications. Forms of carbon are natural choices for the conductive filler. The basic system includes combining carbon black and a non-conductive mechanically flexible matrix to produce a composite. As the content of carbon black increases, the mechanical flexibility of the monofilament decreases, while the electrical conductivity increases. This can result in a monofilament which conducts current well, but will fail mechanically if stressed too much. Conversely, if greater mechanical performance of the monofilament is desired (large strain range), it may come at the expense of the electrical properties.

The relationship between filler concentration and conductivity is explained using the percolation theory [25]. As the concentration of conductive filler particles in an insulating matrix increases, a point will be reached where the electrical resistance will drop dramatically, signifying that a continuous conductive network has been achieved within the material. For CPCs based on carbon black, the percolation threshold is strongly influenced by particle and aggregate geometry [26]. For a strain sensor, the monofilament must be able to undergo high mechanical deformation, ideally between 20%–100%, in order to sufficiently outperform conventional strain sensors based on thin wires integrated into a strain sensor design.

One of the piezoresistive monofilament systems described in this article is composed of CB mixed with a TPE matrix. Previous work at Empa (Dübendorf, Switzerland) and ETH Zurich [27] investigated the ratio of carbon black to SEBS in the monofilament system, it was determined that a 50% weight combination of SEBS and carbon black produced a stable system with good electrical and mechanical performance. Specific applications included the integration of monofilaments into a form-fitted shirt which could be used to identify the body position of a person wearing the shirt [28]. Sensors based on conductive polymers are available commercially, and were considered for this application.

### 1.4. Commercial Sensors

Currently, the primary focus of commercially produced electrically conductive polymer sensors is for force sensing. Commercially available flat force sensors are generally composed of carbon filled polymers or conductive ink layers with electrodes on the top and/or bottom. For example, the TekScan FlexiForce^®^ sensors rely on the compression of carbon-filled polymer material sandwiched between parallel electrodes, the application of force perpendicular to the sensor surface leads to a decrease in resistance of the polymer layer [29]. This occurs because compression pushes the carbon particle closer together, allowing more connections through the conductive network. Also common is the force-sensing resistor (FSR) design, which includes a layer of conductive ink or polymer, then an air gap and a set of interdigitated electrodes [30,31]. The FSR design does not rely on the mechanical coupling between the polymer and the applied strain, but rather as an increasing vertical force is applied to the sensor, an increasing number of the electrodes contact the conductive layer and, the overall resistance drops, which can then be correlated to the applied force. Both of these sensor types are easily obtained commercially, but are only useful to sense applied force perpendicular to the surface of the sensor. These sensors mainly use Mylar as a substrate material, making them robust, but inflexible to stretch deformation. Different types of conductive polymer monofilaments are also available, offering the possibility of a high-stretch variable resistance sensor.

High-stretch monofilament fiber sensors are commercially available from Images SI Inc., and previously from Merlin Systems Corp. Ltd. (Plymouth, UK). The Merlin fibers, composed of carbon impregnated rubber, have been investigated by other researchers for human computer interaction (HCI) applications of the direct control of musical performances, and compared with custom-designed knitted conductive fiber sensors [32]. The Merlin fibers, used as variable resistors, were integrated into Powernet (a synthetic stretch fabric). The panels were oriented at high-stretch areas near the elbows and across the back and upper arm. The variable analogue sensor input from the fibers was digitized with an Arduino (using Arduino-to-Max) to create MIDIs for the electronic music software Ableton [33]. In this work it was found that the first upper and lower limits of the fiber signals were different than subsequent cycles, which were more stable [32]. Images SI conductive monofilament fibers are composed of EA60 silicone elastomer platinum-cured polysiloxane and a conductive filler [34].

### 1.5. Current Work

Piezoresistive polymer monofilament sensors offer a way to produce flexible strain sensors at an affordable price point. The current work investigates the strain sensor characteristics of commercially available monofilaments and of a CB/TPE composite monofilament for integration into a wearable computing application. Tensile and cyclic tensile straining of the monofilaments was done to characterize the mechanical and electrical response to applied mechanical strains and assess any changes in signal drift due to multiple loadings.

## 2. Experimental Section

Monofilament fibers were obtained from Merlin Inc. and Image SI Inc. for testing, while CB/TPE monofilaments were produced at Empa. The Merlin and Image SI monofilaments had nominal diameters of 2.0 and 1.72 mm. Empa monofilaments were produced with 2.0 and 0.30 mm diameters, starting from the base materials and then meltspinning the monofilaments (monofilaments).

### 2.1. Feedstock Mixing and Materials

For the Empa monofilaments, Styrene-ethylene/buthylene-styrene (SEBS) triblock copolymer, produced by KRAIBURG TPE GmbH & Co. KG (Waldkraiburg, Germany) was used as the matrix and carbon black produced by TIMCAL Graphite & Carbon, (Bodio, Switzerland) was employed as the filler. The carbon black particles had a density of 1.86 g/cm^3^ (measured by Helium Pycnometer) and a specific surface area of 61.48 m^2^/g (BET Nitrogen Surface Area measurement). The particle size distributions for carbon black are shown in Table 1. The theoretical particle size based on the surface area measurement was 548 nm.

The materials were mixed together using a torque rheometer (HAAKE Polylab OS, equipped with a HAAKE Rheomix OS high mixer chamber, Thermo Electron Corporation (Waltham, MA, USA) and type 600 Roller rotors. The chamber temperature was controlled electronically by independent heating and cooling zones. Before mixing, the carbon powder was dried in the oven for 1 h at 120 °C. The three zones of the mixing chamber were heated up to 180 °C and rotation was set to 10 rpm. The polymer was filled in and mixed for five minutes, before the carbon black was added. The compounds were then mixed for approximately 1 h until the torque reached steady-state conditions.

### 2.2. Monofilament Meltspinning

Monofilaments were produced by extruding the feedstock material using a Rosand RH7 Flowmaster capillary rheometer (Malvern Instruments Ltd., Malvern, UK). A Niggeloh 200 bar pressure transducer was used to monitor the pressure of the melt through an orifice with a diameter of 300 μm to shape the extruded material into monofilament form. The heating zones of the chamber were set to 190 °C and the feedstock material was placed into the bore and left there for 30 min to melt. A shear rate of 1,500/s were used to manufacture the monofilaments.

### 2.3. Mechanical Testing

Cyclic tensile loading was used to investigate the electrical-mechanical property coupling of the monofilaments. A Zwick Z005 tensile testing machine with ZwickRoell 500N and X Force P 200N load cells (Zwick, Ulm, Germany) and testExpert v11.1 software (Zwick, Ulm, Germany) was used to apply mechanical load, while a Metex M-3610D multimeter (Metex Corporation, Seoul, Korea) was used to record the resistance of the monofilament in response to applied mechanical strain. ScopeView software (Metex Corporation, Seoul, Korea) was used with the multimeter to log the resistance measurements on a laptop computer.

**Figure 1.**
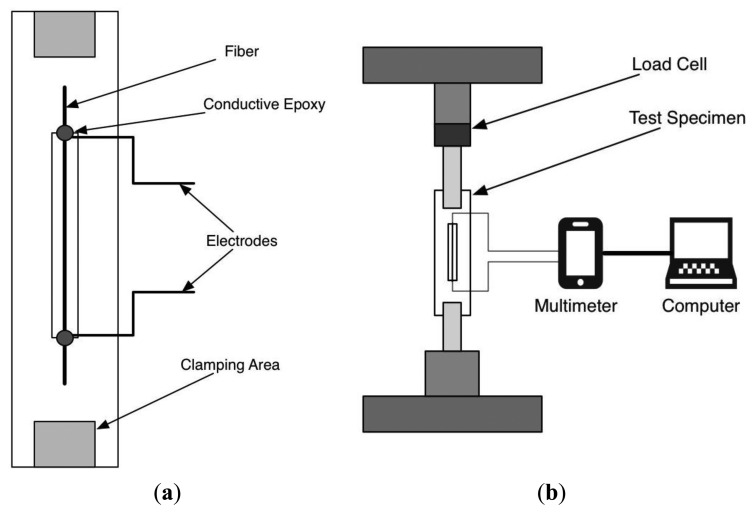
(**a**) Tensile specimen test frame with monofilament position and electrodes; (**b**) Tensile test setup, including testing machine and resistance signal monitoring.

Test samples with a 50 mm gage length were prepared using standard monofilament testing frames [35]. Carton frames with a total length of 120 mm and width of 30 mm (with a window 50 mm in length cut in the center) were used to mount the monofilaments. A line along the frame was used as a guide, and double-sided tape was used to temporarily secure the monofilaments in the correct position. Quick-drying glue was applied to affix the monofilament to the carton, with a little space at the top and bottom of the monofilament to apply electrical connections. Silver-coated copper wires with a length of 50 cm were gently placed in position and conductive silver epoxy CW2400-A and -B (ITW Chemtronics, Kennesaw, GA, USA) was used to connect the wires to the monofilaments, which were then connected to the multimeter as shown in Figure 1.

Tensile testing to failure was done to observe the linearity of the resistance signal. To assess the strain sensor properties, tensile cyclic testing was used. The cycle tests and monofilament types are listed in Table 2, where each strain level was held for 60 s, and five consecutive cycles between the high and low values were employed in each test. The cycling shows how each sensor reacted to repeated strains, and allows the investigation of relaxation and drift effects of the mechanical as well as electrical signals.

## 3. Results and Discussion

The monofilaments were compared to one another based on their resistance values, sensitivity to applied strain, and signal drift when held at specific strain levels. The base resistance values give an indication of filler content while mechanical loading through cycle testing shows differences in sensor sensitivity. Relaxation here is defined as a decrease of stress over time under a constant deformation while drift is defined as the change in resistance over time when a particular strain is applied. The tensile loading and cycle experiments also showed the base properties as a strain sensor of the different monofilaments.

### 3.1. Resistance Values

Resistance of the monofilaments was measured in the unstrained state to establish the base values. Equation (2) was used to calculate resistivity (*ρ*), where *R* is electrical resistance (Ω), *A* is cross-sectional area, and *l* is the length of the material:

(2) $$\rho =R\;\frac{A}{l}$$

The Ω/mm and the Ω·mm values were calculated and are listed in Table 3, where 50 mm is the uniform length for each monofilament to allow a direct comparison. The Merlin and Images SI monofilaments displayed similar resistance values, while the 2.0 mm Empa monofilament has much lower resistance. The 0.30 mm Empa monofilament retained the highest resistance at 36 Ω/mm which can be explained by the smaller cross section.

### 3.2. Tensile Loading Results

**Figure 2.**
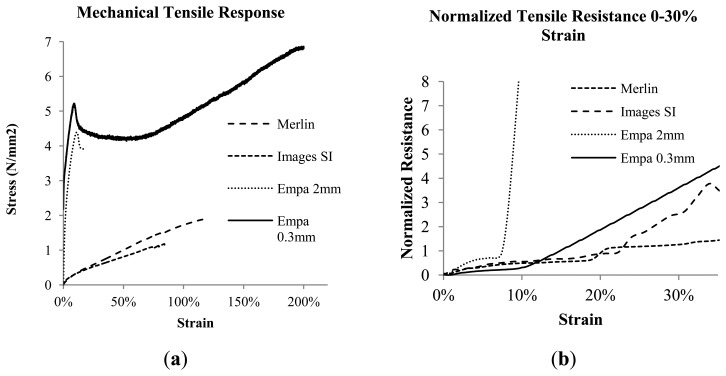
(**a**) Stress-strain tensile response of the monofilaments and (**b**) normalized resistance-strain response from 0%–30% strain.

The tensile loading response of the monofilaments is shown in Figure 2a, where the Merlin and Images SI monofilaments show a non-linear loading profile characteristic of silicone or natural rubber composite materials [36], while the Empa monofilaments show a yield point, followed by subsequent relaxation at higher strains, more characteristic of thermoplastic materials. The tensile resistance-strain response of the monofilaments is shown in Figure 2b, where the Empa 0.3 mm monofilament shows the highest resistance as strain increases, and the slope change near 15% corresponds with the yield point of the material (Figure 2a). The Empa 0.3 mm monofilament can achieve a high maximum strain as compared with the Empa 2 mm monofilament. There were grip failure problems with the thick monofilaments, including the Merlin and Images SI specimens. The monofilaments failed near the grips and some debonding occurred to the glue securing the monofilament to the test frame and also there was cracking of the conductive epoxy, which needs to be known when interpreting the sensor response at high levels. This was not a problem however, with the cycle testing of monofilaments in the 15%–30% range.

In order to compare the different materials to one another, normalized resistance was calculated based on the resistance at 0% strain for each monofilament type according to Equation (3), where *R*_0_ is the resistance at a strain of zero, and *R_m_* is the measured resistance at a particular strain:

(3) $$R=\frac{\left(R_{m}-R_{0}\right)}{R_{0}}$$

The resistance-strain response of the monofilaments is shown in Figure 2b, where the Merlin monofilament shows a lower slope than the Merlin and Images SI monofilaments. The resistance signal for the Merlin and Images SI monofilaments was erratic at points above 30% strain, and may have occurred due to cracking of the conductive epoxy at the electrical connections with contraction of the material geometry at higher strains (this is not an issue with the 0.3 mm Empa monofilaments). The Images SI monofilament shows a near-linear response from 0%–23% strain, where a slope transition is seen and slightly higher slope beyond that. The Empa 2 mm monofilament failed at a significantly lower strain than the other monofilaments, reaching a maximum strain of 10%–15% on average. Failure of the Empa 2 mm monofilaments occurred as necking near the grips and then rupture at that point. Even by using roller grips instead of the paper frame the failure at the grips could not be avoided. The use of roller grips improved the maximum strain by a few percent, but was not a significant improvement. This behavior differs from the Empa 0.3 mm monofilament, where mechanical yielding was very uniform along the length of the monofilament during loading, and failure generally occurred in the middle of the monofilament. The resistance-strain response of the Empa 2 mm monofilament is rather low compared to the other monofilaments, and shows a slope transition near 8%, also corresponding the mechanical yield point, as was seen with the Empa 0.3 mm monofilaments.

### 3.3. Cycle Testing Results

**Figure 3.**
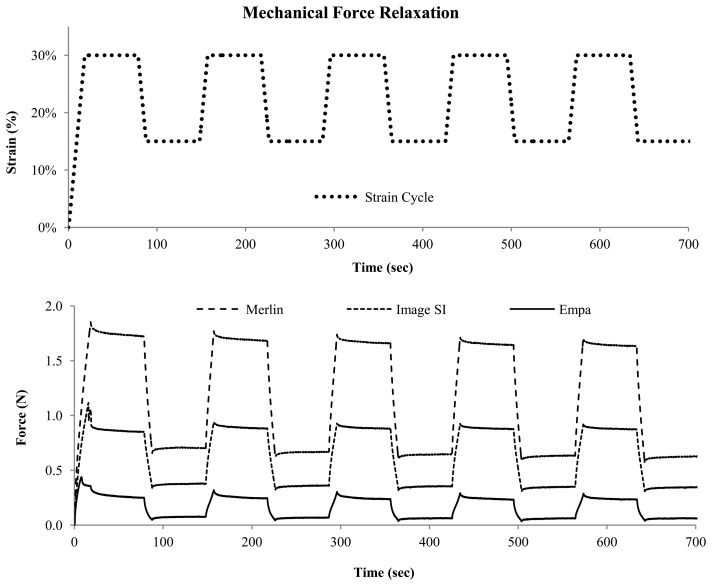
Mechanical response of the monofilaments, loaded to 30%, and then cycled between 15% and 30% strain levels.

When considering the application of wearable sensors to track body position or form, it is clear that tight fitting textiles are required, and that pre-strain of the piezoresistive monofilaments will be needed. Therefore cycle behavior was investigated with a certain pre-strain. After loading to 30%, the monofilaments were cycled between 15%–30% for four cycles and the resistance was tracked during this time. Signal relaxation and drift could then be evaluated by looking at the loading and unloading profiles of the signal. The Force relaxation of the Empa 0.3 mm, Merlin, and Images SI monofilaments are shown in Figure 3, where a mechanical relaxation is seen in the initial loading to 30% strain for all monofilaments. This peak is much less pronounced in the second cycle, and the relaxation behavior stabilizes by the third or fourth cycle.

**Figure 4.**
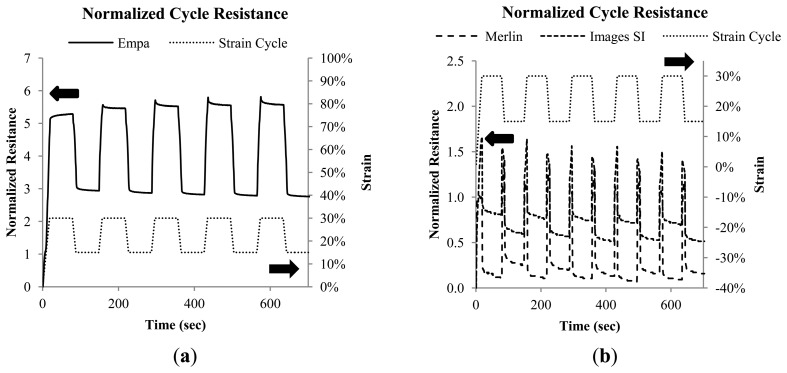
(**a**) Cyclic (15%–30%) tensile loading behavior of the Empa 0.3 mm monofilaments and (**b**) behavior of the Images SI, and Merlin monofilaments, with strain cycle included for reference.

Figure 4a shows the strain loading profile as well as the resistance response of the Empa 0.3 mm monofilaments. Figure 4b shows the signal response for the Merlin and Images SI monofilaments. The Merlin and Images SI monofilaments show similar cycle behavior, with Images SI having a slightly higher signal. The difference in normalized resistance between 15% and 30% is 0.21 Ω/Ω for Images SI, and 0.14 Ω/Ω for the Merlin monofilament. Both monofilaments show a large signal peak when transitioning between strain levels. The signal of the Images SI and Merlin monofilaments increases dramatically when transitioning between strain levels and the resulting signal peak must stabilize at each new strain level. This is a barrier to using the Images SI or Merlin monofilaments for continuous strain monitoring, and makes them more suitable to tracking state changes in an on/off configuration. By comparison, the Empa 0.3 mm monofilaments show good adherence to the applied strain level, both while transitioning between and when holding at the static strain levels, with only small signal peaks (relaxation) when transitioning between dynamics and static strain states. The Empa 0.3 mm monofilament also shows a slight increase in signal level from the first to the last strain cycle. This is reflective of the need to pre-strain the monofilaments before using them in an application. The 0.3 mm Empa monofilament has a resistance difference of 2.79 Ω/Ω between the 15%–30% strain steps.

The theory of conductivity in conductive polymers generally assumes that the individual particles touch at different points in the polymer matrix, enabling conductive pathways through the material [37]. As mechanical strain is applied, the separation between conductive particles increases, leading to a decrease in the conductivity of the material. On the other hand, at large filling content new pathways are generated because of the rotation of the structured nanoparticles [37]. These models can explain the resistance behavior in all the monofilaments and have been studied previously [37]. The Merlin monofilament displays a lower resistance at higher strain levels. For example, the resistance at 30% strain is 0.85 kΩ and 0.92 kΩ at 15% strain. Similar results has been also investigated for the Empa monofilaments with low carbon black content and is reported in [11,27]. The lower resistance could explained by the alignment of conductive particles, which changes with strain and orientation along the monofilament length, leading to an increase in connections in the internal conductive particle networks, and a decrease in conductivity.

**Figure 5.**
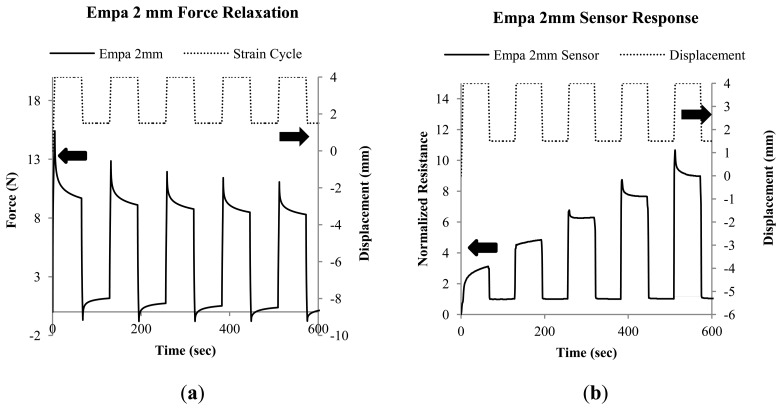
(**a**) Mechanical relaxation of the Empa 2 mm monofilaments and (**b**) resistance signal relaxation during mechanical cycling between 2%–8% strain.

Figure 5a shows the force response and Figure 5b shows the resistance signal response of the 2 mm Empa monofilaments (cycled between 2%–8% to reduce the possibility of tensile failure during the test). Here a very pronounced resistance increase with successive loadings is seen, and also the development of loading peaks after the third cycle exists. The change in resistance suggests that a reorientation of the conductive pathways in the material occurred within in the first loading cycles. This behavior is also seen with Empa 0.3 mm specimens (Figure 5a), but not to such a large extent. The 2 mm results also illustrate well a decoupling between mechanical force and resistance signal relaxation. The mechanical signal response stabilizes by the third cycle, but the resistance continues to change through the fifth cycle.

Table 4 displays the gauge factor (*k*) values for the 15% and 30% strain steps, which were calculated according to Equation (1) in order to better characterize the strain gauge performance. The resistance values at the end of each strain step were used in the calculation to reflect the stabilized measurement value. The gauge factor was lower at 30% than at 15% strain for both the Merlin and Images SI monofilaments, while the Empa 0.3 mm showed relatively stable performance between both strain steps. The Empa 0.3 mm monofilament attained a gauge factor of over 18 for both strain steps, and the difference in average gauge factor between the 15% and 30% strain steps was only 3.4%. The Merlin monofilament showed the poorest performance at 30% strain of 0.32, while the Images SI monofilament achieved gauge factors of 2.48 and 3.62 at 30% and 15% respectively, which is comparable to conventional metal foil strain gauges.

### 3.4. Sensor Characteristics

**Figure 6.**
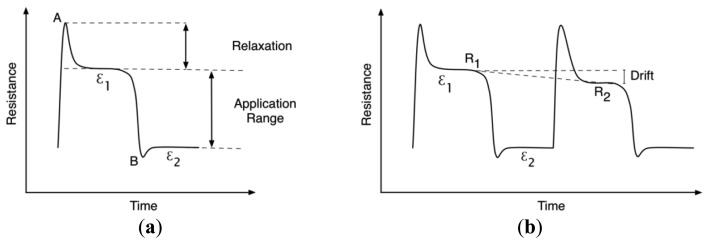
Definition of signal relaxation (**a**) and drift (**b**) behavior for cyclic tensile measurements.

The monofilaments were compared as strain sensors, with attention paid to the change in signal relaxation, drift, and resolution over the cycle tests. Drift was characterized as a decrease in resistance between the initial 30% strain and successive strain steps, while signal relaxation characterizes the decrease in resistivity in each individual strain level while the monofilament is held at each strain step. Figure 6a shows an idealized curve for a piezoresistive polymer under tensile loading, where points A and B represent the peaks which occur when transitioning from a dynamic to a static strain state. The relaxation is then the difference between the initial peak and the relaxed signal state (where the signal has stabilized). The strain application range is the difference between ε_1_ and ε_2_. Figure 6b shows the drift definition, which is defined as the difference between *R*_1_ and *R*_2_ divided by *R*_1_. Drift results were computed from the Images SI, Merlin, and Empa 0.3 mm monofilaments.

**Figure 7.**
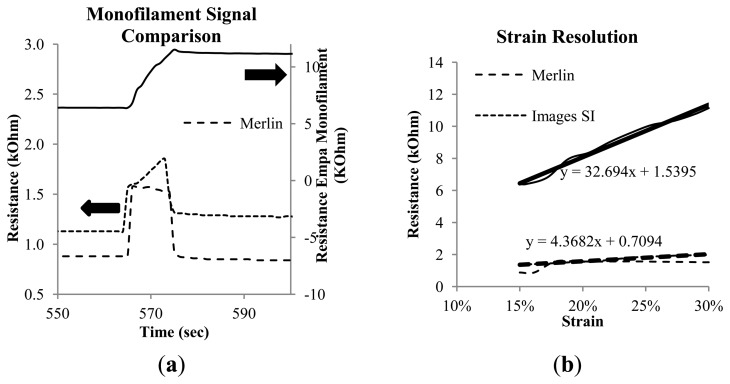
(**a**) Signal relaxation and drift (**b**) results at the 30% strain level.

The relaxation results are shown in Figure 7a for the 30% strain level and the signal drift results are shown in Figure 7b. As expected, all monofilaments showed signal relaxation behavior, however the Empa 0.3 mm monofilaments showed the least amount (Figure 7a) over the five cycles, stabilizing at 3.5% for the 30% strain step. A relation for this monofilament below 8% was already reported by Mattman *et al.* [11]. The first cycle at 30% of the Empa 0.3 mm is negative since a slight increase in resistance occurred, similar to the behavior of the 2 mm Empa monofilament (Figure 7b). This is indicative of the material yield point around 10%–15%, and that conditioning (pre-straining) of the material is needed before use. The signal drift results in Figure 7b show that drift fluctuates for the Merlin monofilaments, reaching levels of −3.0% to 3.5% at the 4th and 5th cycles respectively. The Images SI monofilament shows better drift performance, with a maximum of 2% at the second cycle. The Empa 0.3 mm showed an initially high drift at the second cycle, which then falls below 1% by the 5th cycle.

**Figure 8.**
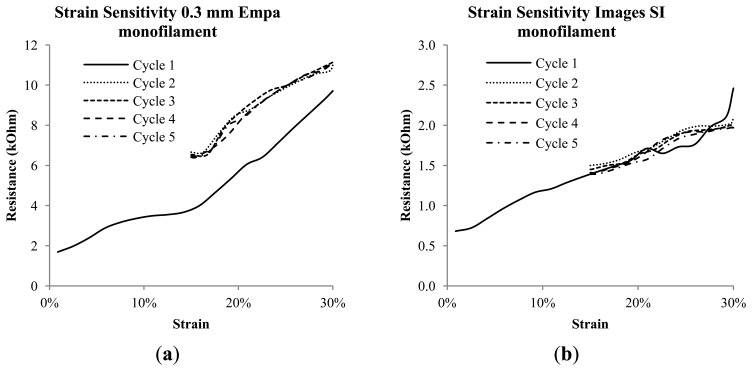
(**a**) Last strain step from 15%–30% of the cycle testing and (**b**) the corresponding resistance *vs.* strain plot with slopes calculated for the Images SI and Empa monofilaments.

**Figure 9.**
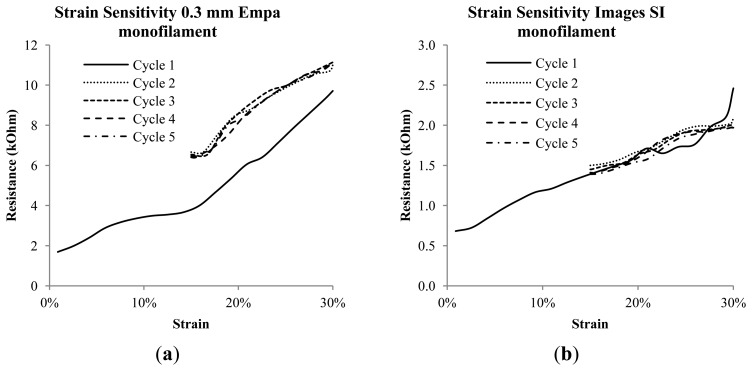
(**a**) Successive loadings of the cycle testing of the Empa 0.3 mm and (**b**) Images SI monofilaments.

In Figure 8a the last strain step from the cycle testing is shown, and Figure 8b depicts the corresponding resistance *vs.* strain curves. Here treadlines are added to show the strain sensing resolution of the Images SI and Empa 0.3 mm monofilaments. The Merlin monofilament does not accurately track the changing strain, having an essentially flat response to the mechanical loading beyond the initial and abrupt rise due to the changing strain state. The Images SI monofilaments display lower resolution per strain (4.4 kΩ/%) than the Empa monofilament, which has a resolution of 32.7 kΩ/%. Figure 9 shows the cyclic loading results from 0%–30% and 15%–30% for the Empa 0.3 mm (a) and Images SI (b) monofilaments. Both monofilaments show some non-linear but repeatable behavior after the first cycle. This more clearly shows the need for conditioning or pre-straining of the Empa monofilament before obtaining a repeatable signal. The Images SI monofilaments show good agreement from the first to the last cycle, however the transition from one strain step to another is not very smooth as seen in Figure 8a.

## 4. Conclusions

All of the monofilaments were able to determine different stain levels during cycle testing, and within their capacities, could be used as strain sensors, but differences were observed between the different monofilament types. During transitions between static and dynamic strain states, large signal spikes were seen with the Images SI and Merlin monofilaments, making them less desirable for continuous strain monitoring applications. Signal relaxation and drift of the sensor signal while holding at specific strain levels was present, and after successive strain cycling, the Empa 0.3 mm monofilament showed the lowest signal relaxation and drift values. The Images SI and Empa 0.3 mm monofilaments provided a near-linear response when loading between 15%–30%, however, the Merlin monofilaments showed a signal plateau after 16%–18% strain. The 2 mm Empa monofilaments composed of carbon black and a thermoplastic elastomer failed at low strains due to stress concentrations at monofilament clamping (necking points due to mechanical yielding), making the 2 mm Empa monofilaments undesirable for high-strain sensing applications. The Empa 0.3 mm monofilaments provided a high signal response at different strain steps, but also allowed good continuous monitoring of strain transition stages between strain steps. Mechanical yielding of the 0.3 mm Empa monofilaments occurs along the monofilament length, which enables a high ultimate strain (by reducing the material necking and subsequent stress concentration seen with the Empa 2 mm monofilaments), and allowing the monofilament to be used in high strain applications (>100%). However, pre-straining is needed before using the Empa monofilaments due to material yielding at strains of 10%–15%. Pre-straining stretches the material beyond the mechanical yield point, which is dominated by the thermoplastic component of the TPE. Beyond this yield point, the elastomer component of the material allows for high strains. Furthermore, signal drift is reduced, providing a more stable sensor response. The combination of high signal resolution, high failure strain, and low signal relaxation and drift make the 0.3 mm Empa monofilaments a stronger candidate than the other tested monofilaments for wearable computing applications where continuous monitoring at high strains is required.

## Figures and Tables

**Table 1.** Particle size distributions for the carbon black particles.

d_10_ (μm)	0.256
d_50_ (μm)	0.622
d_90_ (μm)	2.023

**Table 2.** Listing of tensile and cyclic strain tests for the different monofilament types and their nominal expected thicknesses. Due to their low failure strain, the 2 mm Empa monofilaments were only cycled between 2% and 8% strain due their lower failure limit.

**Monofilaments**	**Diameter (mm)**	**Cycle**
Images SI	1.72	15%–30%
Merlin Systems	2.00	15% –30%
Empa	2.00	2% –8%
Empa	0.30	15% –30%

**Table 3.** List of monofilament, their nominal expected thicknesses, and their resistance values, based on 50 mm length.

**Monofilament Type**	**Dia. (mm)**	**Ω/mm**	**Ω·mm**
Images SI	1.72	13.62	31.65
Merlin Systems	2.00	15.20	47.75
Empa	2.00	1.03	3.01
Empa	0.30	36.00	2.54

**Table 4.** Gauge factor (*k*) values for the different monofilaments at 30% and 15% strain steps.

**30% Strain**						

Cycle	1	2	3	4	5	Avg.
Merlin	0.35	0.35	0.35	0.22	0.31	0.32
Images SI	2.66	2.52	2.48	2.39	2.34	2.48
Empa 0.3 mm	17.61	18.19	18.40	18.50	18.54	18.25

**15% Strain**						

Merlin	1.67	1.32	0.88	1.05	1.05	1.19
Images SI	3.96	3.78	3.42	3.51	3.42	3.62
Empa 0.3 mm	19.61	19.13	18.82	18.58	18.38	18.90
